# Safety and immunogenicity of inactivated SARS-CoV-2 vaccine in high-risk occupational population: a randomized, parallel, controlled clinical trial

**DOI:** 10.1186/s40249-021-00924-2

**Published:** 2021-12-22

**Authors:** Yongliang Feng, Jing Chen, Tian Yao, Yue Chang, Xiaoqing Li, Rongqin Xing, Hong Li, Ruixue Xie, Xiaohong Zhang, Zhiyun Wei, Shengcai Mu, Ling Liu, Lizhong Feng, Suping Wang

**Affiliations:** 1 Department of Epidemiology, School of Public Health, Shanxi Medical University, 56 Xinjian South Road, Taiyuan, 030001 Shanxi Province China; 2 Center of Clinical Epidemiology and Evidence Based Medicine, Shanxi Medical University, Taiyuan, China; 3 Shanxi Provincial Center for Disease Control and Prevention, 8 Xiaonanguan Street, Taiyuan, 030012 Shanxi Province China; 4 Shanxi Provincial Key Laboratory for Major Infectious Disease Response, Taiyuan, China; 5 Outpatient Department of Shanxi Aviation Industry Group Co. LTD, Taiyuan, China

**Keywords:** COVID-19, Inactivated SARS-CoV-2 vaccine, Immunogenicity, Safety, High-risk occupational population, Randomized controlled trial

## Abstract

**Background:**

Severe acute respiratory syndrome coronavirus 2 (SARS-CoV-2) infection and the resulting coronavirus disease 2019 (COVID-19) have a substantial burden on health-care systems around the world. This is a randomized parallel controlled trial for assessment of the immunogenicity and safety of an inactivated SARS-CoV-2 vaccine, aiming to determine an appropriate vaccination interval of the vaccine for high-risk occupational population.

**Methods:**

In an ongoing randomized, parallel, controlled phase IV trial between January and May 2021 in Taiyuan City, Shanxi Province, China, we randomly assigned the airport ground staff and public security officers aged 18 to 59 years to receive two doses of inactivated SARS-CoV-2 vaccine at 14 days, 21 days, or 28 days. The serum neutralizing antibody to live SARS-CoV-2 was performed at baseline and 28 days after immunization. Long-term data are being collected. The primary immunogenicity endpoints were neutralization antibody seroconversion and geometric mean titer (GMT) at 28 days after the second dose. Analysis of variance (ANOVA), chi-square, and logistic regression analysis were used for data analysis.

**Results:**

A total of 809 participants underwent randomization and received two doses of injections: 270, 270, 269 in the 0–14, 0–21, and 0–28 vaccination group, respectively. By day 28 after the second injection, SARS-CoV-2 neutralizing antibody of GMT was 98.4 (95% *CI:* 88.4–108.4) in the 0–14 group, which was significantly lower compared with 134.4 (95% *CI*: 123.1–145.7) in the 0–21 group (*P* < 0.001 vs 0–14 group) and 145.5 (95% *CI*: 131.3–159.6) in the 0–28 group (*P* < 0.001 vs 0–14 group), resulting in the seroconversion rates to neutralizing antibodies (GMT ≥ 16) of 100.0% for all three groups, respectively. The intention-to-treat (ITT) analysis yielded similar results. All reported adverse reactions were mild.

**Conclusions:**

Both a two-dose of inactivated SARS-CoV-2 vaccine at 0–21 days and 0–28 days regimens significantly improved SARS-CoV-2 neutralizing antibody level compared to the 0–14 days regimen in high-risk occupational population, with seroconversion rates of 100.0%.

***Trial registration*:**

Chinese Clinical Trial Registry, ChiCTR2100041705, ChiCTR2100041706. Registered 1 January 2021, www.chictr.org.cn.

**Graphical Abstract:**

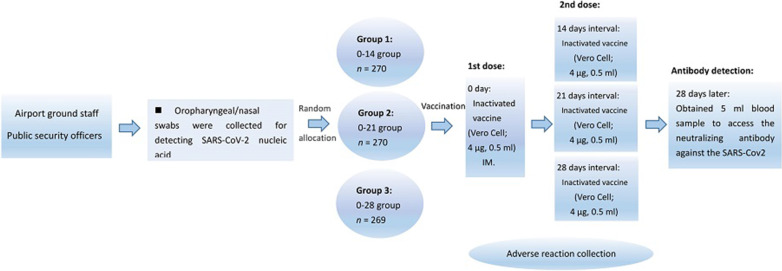

**Supplementary Information:**

The online version contains supplementary material available at 10.1186/s40249-021-00924-2.

## Background

The ongoing pandemic of coronavirus disease 2019 (COVID-19) induced by severe acute respiratory syndrome coronavirus 2 (SARS-CoV-2) has led to an unprecedented global public health crisis. Globally, as of 26 October 2021, more than 243 million cases of SARS-CoV-2 infection and more than 4.9 million deaths have been reported [1]. SARS-CoV-2 appears to undergo more rapid transmission and variation [2, 3], and due to the lack of standard treatments, a safe and effective vaccine against COVID-19 is urgently needed to prevent the resurgence of the epidemic.

Inactivated viruses have been traditionally used for vaccine development and such vaccines have been found to be safe and effective for the prevention of diseases caused by viruses like influenza virus and poliovirus [4, 5]. Their long history of use confers some advantages, such as well-developed and mature manufacturing processes, and ease of scaling up production and storage. Inactivated SARS-CoV-2 vaccines have been confirmed to induce high levels of neutralizing antibody titers in mice, rats, guinea pigs, rabbits, and nonhuman primates to provide protection against SARS-CoV-2 [6–8]. Moreover, the results of previous clinical trials on the inactivated SARS-CoV-2 vaccines conducted in several countries showed good neutralizing antibody responses and efficacy against disease caused by COVID-19 [9–13]. To date, two inactivated SARS-CoV-2 vaccines manufactured by the Beijing Institute of Biological Products/Sinopharm (China) and Sinovac Life Sciences/CoronaVac (China) have received conditional marketing approval from China National Medical Products Administration and have been placed on WHO's Emergency Use Listing [14, 15].

Previous studies [12, 13, 16–20] have shown that the three immunization programs (0, 14 procedure, 0, 21 procedure or 0, 28 procedure) induce varying degrees of immune effect, but the optimal interval of injections remains unclear. Furthermore, there is lack of studies on the immunogenicity and safety of inactivated SARS-CoV-2 vaccine in high-risk occupational population. The airport ground staff and public security officers, as front-line workers to respectively responsible for ensuring the operation of international flights and the maintenance of social order, are in close contact with other personnel and face greater occupational risk exposure, leaving them susceptible to further waves of SARS-CoV-2 infection. Therefore, we explored the immunogenicity and safety of the COVD-19 inactivated vaccination schemes at three different intervals of either 14 days, 21 days or 28 days in high-risk occupational population to optimize the inactivated vaccination regimen. We would continue to follow up until months 3, 6, and 12 in the further study.

## Methods

### Study design and participants

We conducted a randomized, controlled phase IV trial of the SARS-CoV-2 inactivated vaccine manufactured by Beijing Biological Products Institute Co., Ltd. between January and May 2021 in Taiyuan City, Shanxi Province, China. Written informed consents were obtained from all participants before enrollment. Eligible participants were airport ground staff and public security officers aged 18–59 years, without previous SARS-CoV-2 vaccination and infection, and negative for SARS-CoV-2 nucleic acid. Exclusion criteria were participants with (1) history or family history of allergy, convulsion, epilepsy, encephalopathy or psychosis; (2) any intolerance or allergy to any component of the vaccine; (3) known or suspected diseases including severe respiratory disease, severe cardiovascular disease, severe liver or kidney disease, medically uncontrollable hypertension (systolic blood pressure ≥ 140 mmHg and diastolic blood pressure ≥ 90 mmHg), complications of diabetes mellitus, malignancy, various acute diseases or acute episodes of chronic disease; (4) various infectious, suppurative and allergic skin diseases; congenital or acquired immunodeficiency; (5) other vaccination history within 14 days before vaccination; (6) a history of coagulation dysfunction, a history of non-specific immunoglobulin injection within 1 month prior to enrollment; acute illness with fever (body temperature > 37.0 °C); and (7) being pregnant or breastfeeding.

The protocol was approved by the Ethics Committee of Shanxi Provincial Center for Disease Control (SXCDCIRBPJ2020056001) and Prevention and was conducted in accordance with the Declaration of Helsinki and Good Clinical Practice. All participants signed a consent form after being informed about the study. The trial was registered with ChiCTR.org.cn (ChiCTR2100041705, ChiCTR2100041706).

### Procedures

A computerized random number generator performed block randomization with a randomly selected block size of 6, and eligible participants were randomly assigned into three groups to receive two doses inactivated SARS-CoV-2 vaccine at the schedule of day 0–14, day 0–21, or day 0–28. Each dose of vaccine containing 4 µg of inactivated SARS-CoV-2 virus antigen was intramuscularly injected into the lateral deltoid muscle of the upper arm. The vaccines used in this study were inactivated vaccine (Vero Cell) produced by Beijing Biological Products Institute Co., Ltd. Demographic information [age, gender, body mass index (BMI), marital status, and education level], influenza vaccination history, smoking, drinking, and chronic diseases were collected via questionnaire investigation.

### Safety assessment

After each dose was vaccinated, the participants were observed for any immediate reaction for 30 min, and local and systemic adverse reactions were collected. Participants were required to record the local adverse events and systemic adverse events on diary cards within 7 days of each injection. Any other unsolicited symptoms were also recorded during a 28-day follow-up period after each injection by spontaneous report from the participants combined with the regular visit. The solicited adverse reactions included local reactions (pain, induration, swelling, rash, flush, and pruritus) and systematic reactions [fever, diarrhea, dysphagia, anorexia, vomiting, nausea, muscle pain (non-vaccination sites), arthralgia, headache, cough, dyspnea, skin and mucosal abnormalities, acute allergic reactions, and fatigue].

### Laboratory methods

Oropharyngeal/nasal swabs were collected for detecting SARS-CoV-2 nucleic acid from all subjects by using reverse transcriptase-polymerase chain reaction (RT-PCR) test to determine whether subjects have occurred SARS-COV-2 infection before the first, second dose of vaccine vaccination, and 28 days after the whole course of vaccination, respectively. Blood samples were taken from participants for serology tests before the first injection and on day 28 after the second injection. The neutralizing antibody to live SARS-CoV-2 [strain 19nCoV-CDC-Tan-Strain 05 (QD01)] were quantified using a micro cytopathogenic effect assay at baseline and 28 days after immunization. A positive antibody response (seroconversion) was defined as post-injection titer of at least 1:16 if the baseline titer was below 1:4, or at least a fourfold increase in post-injection titer from baseline if the baseline titer was at least 1:4 [21]. We defined the neutralizing antibody seroconversion rate as post-injection titer of a 16-fold (Baseline titers were all below 1:4).

### Outcomes

**Fig. 1:**
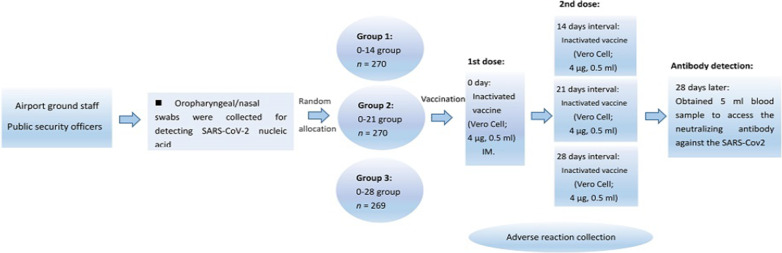
Study protocol

The primary immunogenic endpoints were the seroconversion rates [geometric mean titer (GMT) ≥ 16] and GMT of neutralizing antibody to live SARS-CoV-2 at day 28 after the last dose. Secondary immunogenic endpoints were the positive rates (GMT ≥ 32, 64, 128, 256) 28 days after the whole course of vaccination, respectively. The primary endpoint for safety was the occurrence of adverse reactions within 7 days after the first and second vaccinations. Adverse events within 28 days after the first and the second vaccinations across the three groups were analyzed as secondary safety endpoints. Figure 1 shows the study protocol.

### Statistical analysis

The study sample size of 360 participants provided 84.4% power to detect a difference of 5% (85% vs 80%) of responders in the 0–21 and 0–28 vaccination groups compared with the 0–14 group, respectively in airport ground staff and public security officers. Data were recorded using EpiData version 3.1 (EpiData Association, Odense, Denmark), and analyses were performed using SAS version 9.3 (SAS Institute, Cary, NC, USA). Analysis of variance (ANOVA) was used to analyze continuous data, and the chi-square or Fisher’s exact test was used for categorical data. We assessed immunogenic endpoints by the intention-to-treat (ITT) analysis (i.e., subjects who undertake randomization) and per-protocol (PP) analysis (i.e., subjects who compliant to the protocol, receive 2 doses of vaccine according to the requirements of the protocol, and have serum-testing results before and after immunization). Multinomial logistic regression analysis and unconditional logistic regression model were used to determine the influencing factors of SARS-CoV-2 neutralizing antibody immunization. The safety analysis was performed on data from all subjects who received vaccination after randomization. The level of statistical significance for all analyses was *P* < 0.05.

## Results

### Study participants and baseline characteristics

**Fig. 2:**
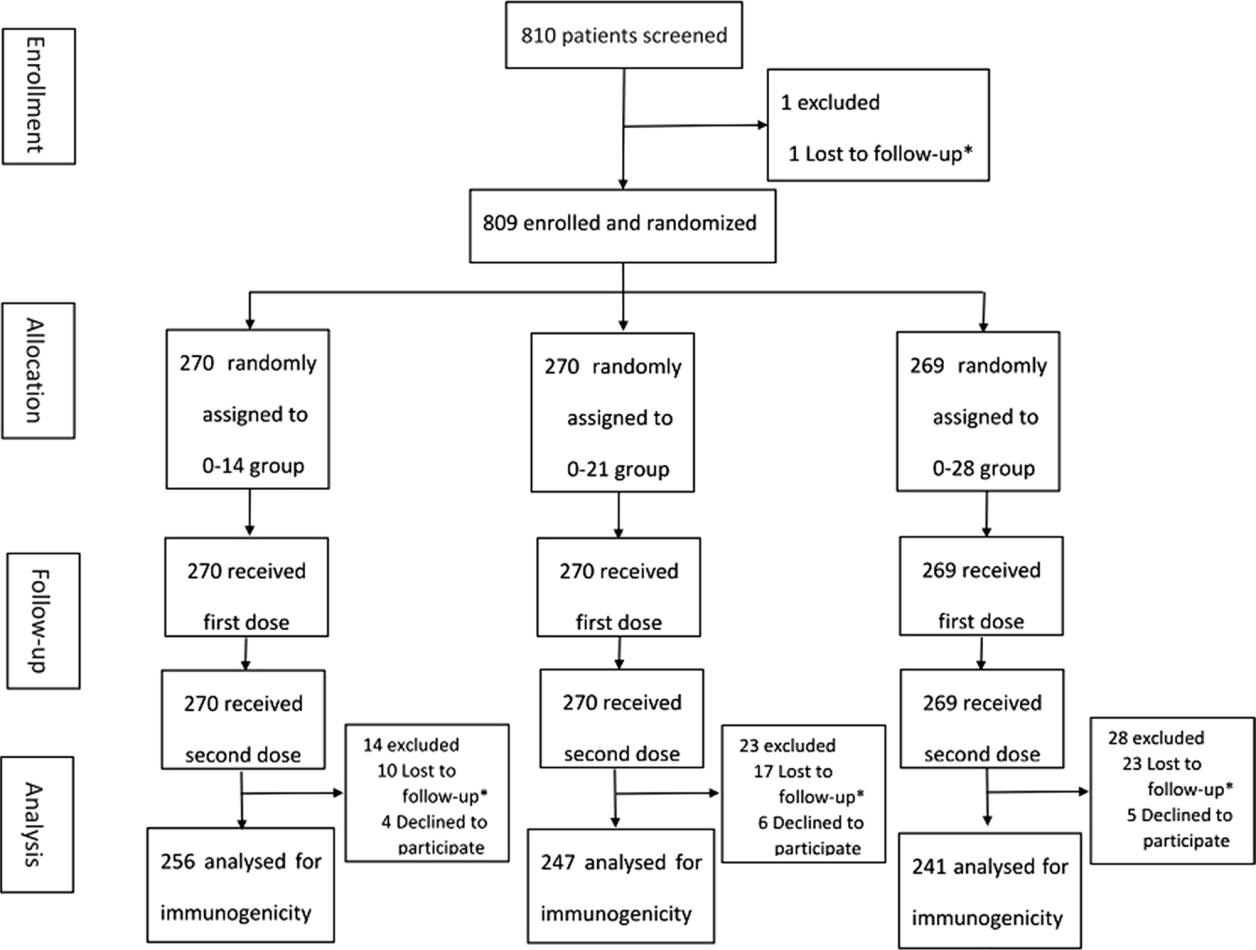
Flow of participants in a study of the inactivated SARS-CoV-2 vaccine in high-risk occupational population. *Lost to follow up including not being at the study site, or illness

Between January and May 2021, 810 participants were screened, and 809 were enrolled (73.2% male, 26.8% female; mean age 38.8 years). Of the 809 participants who were enrolled, 405 participants were the public security officers and 404 participants were the airport ground staff; with 270, 270, and 269 participants in the group 0–14, 0–21, 0–28 vaccination cohort. All enrolled participants received the first injection and completed the two-dose vaccination schedule. A total of 256, 247 and 241 patients in the 0–14, 0–21, and 0–28 groups, respectively, completed the follow-up 28 days after the whole course of vaccination (Fig. 2). The baseline characteristics of the participants are shown in Table 1. There were no significant differences in demographic and behavioral characteristics among the three groups at baseline and 28 days after the whole course of vaccination (*P* > 0.05; Table 1, Additional file 1: Supplement 1).

**Table.1:** Baseline characteristics of high-risk occupational population with different vaccinations

Characteristics	Total (*n* = 809)	0–14 group (*n* = 270)	0–21 group (*n* = 270)	0–28 group (*n* = 269)	*P*
Gender					0.449
Male	592 (73.2)	192 (71.1)	196 (72.6)	204 (75.8)	
Female	217 (26.8)	78 (28.9)	74 (27.4)	65 (24.2)	
Age (years)					0.229
< 40	463 (57.2)	144 (53.3)	156 (57.8)	163 (60.6)	
≥ 40	346 (42.8)	126 (46.7)	114 (42.2)	106 (39.4)	
Education level					0.335
Junior high school or lower	74 (9.1)	31 (11.5)	25 (9.3)	18 (6.7)	
Senior high school	37 (4.6)	10 (3.7)	12 (4.4)	15 (5.6)	
College or higher	698 (86.3)	229 (84.8)	233 (86.3)	236 (87.7)	
Ethnicity					0.366^a^
Han ethnicity	797 (98.5)	268 (99.3)	264 (97.8)	265 (98.5)	
Other	12 (1.5)	2 (0.7)	6 (2.2)	4(1.5)	
Marital status					0.267
Married	621 (76.8)	217 (80.4)	196 (72.6)	208 (77.3)	
Unmarried	165 (20.4)	47 (17.4)	66 (24.4)	52 (19.3)	
Divorced or widowed	23 (2.8)	6 (2.2)	8 (3.0)	9 (3.4)	
BMI (kg/m^2^)					0.848
< 18.5	19 (2.3)	8 (3.0)	6 (2.2)	5 (1.8)	
18.5–	33 3(41.2)	110 (40.7)	116 (43.0)	107 (39.8)	
≥ 24	457 (56.5)	152 (56.3)	148 (54.8)	157 (58.4)	
Influenza vaccination history					0.865
No	549 (67.9)	180 (66.7)	184 (68.2)	185 (68.8)	
Yes	260 (32.1)	90 (33.3)	86 (31.8)	84 (31.2)	
Occupation					0.991
Public security officers	405 (50.1)	135 (50.0)	136 (50.4)	134 (49.8)	
Airport ground staff	404 (49.9)	135 (50.0)	134 (49.6)	135 (50.2)	
Smoking					0.368
No	545 (67.4)	190 (70.4)	181 (67.0)	174 (64.7)	
Yes	264 (32.6)	80 (29.6)	89 (33.0)	95 (35.3)	
Drinking					0.906
No	621 (76.8)	206 (76.3)	206 (76.3)	209 (77.7)	
Yes	188 (23.2)	64 (23.7)	64 (23.7)	60 (22.3)	
Chronic diseases					0.978
No	754 (93.2)	252 (93.3)	252 (93.3)	250 (92.9)	
Yes	55 (6.8)	18 (6.7)	18 (6.7)	19 (7.1)	

Results expressed as *n* (%)

^a^Fisher’s exact test

### Assessment of immunity elicited by the vaccine in the three immunization procedures

#### Seroconversion rate and GMT of SARS-CoV-2 neutralizing antibody in the three groups

By day 28 after the second injection, the seroconversion rates of neutralizing antibody (GMT ≥ 16) were all 100.0% in the 0–14, 0–21, and 0–28 groups. SARS-CoV-2 neutralizing antibody with a GMT of 98.4 (95% *CI*: 88.4–108.4) was noted in the 0–14 group, which was significantly lower compared with 134.4 (95% *CI*: 123.1–145.7) in the 0–21 group (*P* < 0.001 vs 0–14 group) and 145.5 (95% *CI*: 131.3–159.6) in the 0–28 group (*P* < 0.001 vs 0–14 group). The ITT analysis showed similar results that the GMT were between 93.5 and 129.8 in the three groups.

Then we used different criteria to determine the immunization of neutralizing antibody for comparison. The positive rates of neutralizing antibody GMT ≥ 32 were 86.7% (222/256), 96.4% (238/247; *P* < 0.001) and 95.9% (231/241; *P* < 0.001) in the 0–14, 0–21, and 0–28 groups, respectively. The positive rates of neutralizing antibody GMT ≥ 64, 128, and 256 were 60.9% (156/256), 31.6% (81/256) and 9.0% (23/256) in the 0–14 group, 84.6% (209/247), 55.9% (138/247) and 15.0% (37/247) in the 0–21 group, 80.9% (195/241), 51.5% (124/241) and 17.4% (42/241) in the 0–28 group, respectively (Table 2). The positive rates of neutralizing antibody (GMT ≥ 32, 64, 128, or 256) in the 0–21 and 0–28 groups were significantly higher than that in the 0–14 group. The ITT analysis yielded similar results (Table 2).

**Table.2:** The seroconversion rate and GMT of SARS-CoV-2 neutralizing antibody in the three groups

SARS-CoV-2 neutralizing antibody	Per-protocol analysis			Intention-to-treat analysis		
	0–14 group (*n* = 256)	0–21 group (*n* = 247)	0–28 group (*n* = 241)	0–14 group (*n* = 270)	0–21 group (*n* = 270)	0–28 group (*n* = 269)
Primary analysis						
GMT (95%* CI*)	98.4 (88.4–108.4)^a^	134.4 (123.1–145.7)^b^	145.5 (131.3–159.6)^b^	93.5 (83.7–103.3)^a^	122.0 (110.7–133.2)^b^	129.8 (116.2–143.4)^b^
Seroconversion (GMT ≥ 16)						
No, *n* (%)	0 (0.0)	0 (0.0)	0 (0.0)	14 (5.2)	23 (8.5)	28 (10.4)
Yes, *n* (%)	256 (100.0)	247 (100.0)	241 (100.0)	256 (94.8)^a^	247 (91.5)^ab^	241 (89.6)^b^
Crude *OR* (95% *CI*)	–	–	–	1.0	0.6 (0.3–1.2)	0.5(0.2–0.9)
Adjusted *OR* (95% *CI*)	–	–	–	1.0	0.6 (0.3–1.2)	0.5(0.3–1.0)
Secondary analysis						
GMT ≥ 32						
No, *n* (%)	34 (13.3)	9 (3.6)	10 (4.1)	48 (17.8)	32 (11.8)	38 (14.1)
Yes, *n* (%)	222 (86.7)^a^	238 (96.4)^b^	231 (95.9)^b^	222 (82.2)	238 (88.2)	231 (85.9)
Crude *OR* (95% *CI*)	1.0	4.1 (1.9–8.6)	3.5 (1.7–7.3)	1.0	1.6 (1.0–2.6)	1.3 (0.8–2.1)
Adjusted *OR* (95% *CI*)	1.0	4.1 (1.9–8.8)	3.6 (1.7–7.6)	1.0	1.7 (1.0–2.7)	1.4 (0.8–2.2)
GMT ≥ 64						
No, *n* (%)	100 (39.1)	38 (15.4)	46 (19.1)	114 (42.2)	61 (22.6)	74 (27.5)
Yes, *n* (%)	156 (60.9)^a^	209 (84.6)^b^	195 (80.9)^b^	156 (57.8)^a^	209 (77.4)^b^	195 (72.5)^b^
Crude *OR* (95% CI)	1.0	3.5(2.3–5.4)	2.7(1.8–4.1)	1.0	2.5(1.7–3.6)	1.9 (1.3–2.8)
Adjusted *OR* (95% *CI*)	1.0	3.5 (2.7–5.8)	2.7 (1.8–4.1)	1.0	2.5 (1.7–3.7)	1.9 (1.4–2.8)
GMT ≥ 128						
No, *n* (%)	175 (68.4)	109 (44.1)	117 (48.5)	189 (70.0)	132 (48.9)	145 (53.9)
Yes, *n* (%)	81 (31.6)^a^	138 (55.9)^b^	124 (51.5)^b^	81 (30.0)^a^	138 (51.1)^b^	124 (46.1)^b^
Crude *OR* (95% *CI*)	1.0	2.7 (1.9–3.9)	2.3 (1.6–3.3)	1.0	2.44 (1.7–3.5)	2.0 (1.4–2.8)
Adjusted *OR* (95% *CI*)	1.0	2.7 (1.9–3.9)	2.3 (1.6–3.4)	1.0	2.43 (1.7–3.5)	2.1 (1.4–3.0)
GMT ≥ 256						
No,* n* (%)	233 (91.0)	210 (85.0)	199 (82.6)	247 (91.5)	233 (86.3)	227 (84.4)
Yes, *n* (%)	23 (9.0)^a^	37 (15.0)^b^	42 (17.4)^b^	23 (8.5)^a^	37 (13.7) ^ab^	42 (15.6)^b^
Crude *OR* (95% *CI*)	1.0	1.8 (1.0–3.1)	2.1 (1.2–3.7)	1.0	1.7 (1.0–3.0)	2.0 (1.2–3.4)
Adjusted *OR* (95% *CI*)	1.0	1.8 (1.0–3.2)	2.3 (1.3–4.0)	1.0	1.8 (1.0–3.1)	2.1 (1.2–3.6)

– No result value

*GMT* Geometric mean titer; *CI* Confidence interval; *OR* Odds ratio

^a,b^There was significant difference with the different letters

#### Distribution of SARS-CoV-2 neutralizing antibody in the three groups

**Fig. 3:**
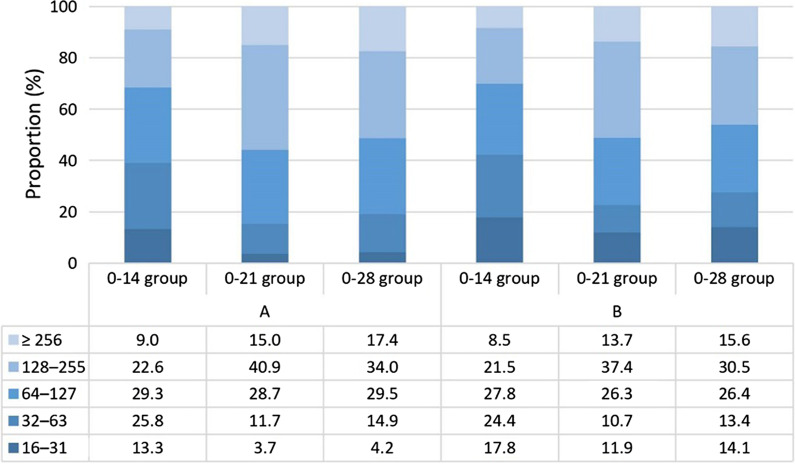
Distribution of SARS-CoV-2 neutralizing antibody. **A** Per-protocol analysis, **B** intention-to-treat analysis. The table shows the percentages of SARS-CoV-2 neutralizing antibody in each group

In the 0–14 group, the proportion of neutralizing antibody was higher at the GMT 32–63 (25.8%, 66/256) and 64–127 (29.3%, 75/256), while the proportion of neutralizing antibody in the 0–21 and 0–28 groups was higher at the GMT of 64–127 (28.7%, 71/247; 29.5%, 71/241) and 128–255 (40.9%, 101/247; 34.0%, 82/241), respectively (Fig. 3). There was a significant difference in distribution of SARS-CoV-2 neutralizing antibody GMT among the three vaccination groups (*P* < 0.001).

#### Stratified analysis

In the further analysis stratified by age and gender, we observed the similar results that the GMT of SARS-CoV-2 neutralizing antibody and positive rates in the 0–21 and 0–28 groups were superior to the 0–14 group for the participants with different characteristic levels (Additional file 1: Supplement 2, Supplement 3). And the similar distribution results of SARS-CoV-2 neutralizing antibody were observed for the three groups (Additional file 1: Supplement 4).

### Influencing factors of SARS-CoV-2 neutralizing antibody immunization by multinomial logistic regression

Multinomial logistic regression was performed to examine the influencing factors of SARS-CoV-2 neutralizing antibody immunization depending on their extent (GMT: 16–63 = 1, GMT: 64–127 = 2, GMT: ≥ 128 = 3, ref = 1) and the results showed that only the vaccination regimen was associated with the antibody response. After adjusting for age, gender, BMI, marital status, education level, influenza vaccination history, smoking, drinking, and chronic diseases, participants who received 0–21 vaccination regimen was 2.5 times higher than the 0–14 vaccination group when GMT was 64–127 (95% *CI*: 1.5–4.1), and it was 4.4-fold higher than that in 0–14 vaccination group at GMT ≥ 128 (95%* CI*: 2.8–7.1). Participants in the 0–28 group had 2.0-fold odds when GMT was 64–127 (95% CI 1.2–3.3) than that in the 0–14 group and had 3.3-fold odds of GMT ≥ 128 (95% *CI*: 2.1–5.2) (Table 3).

**Table.3:** Influencing factors of SARS-CoV-2 neutralizing antibody immunization by multinomial logistic regression

Variables	*OR* (95%* CI*)	*OR* (95% *CI*)*
GMT 64–127 (ref: 16–63)		
0–14 group	1.0	1.0
0–21 group	2.5 (1.5–4.1)	2.5 (1.5–4.1)
0–28 group	2.1 (1.3–3.3)	2.0 (1.2–3.3)
GMT ≥ 128 (ref: 16–63)		
0–14 group	1.0	1.0
0–21 group	4.5 (2.8–7.1)	4.4 (2.8–7.1)
0–28 group	3.3 (2.1–5.2)	3.3 (2.1–5.2)

*GMT*, Geometric mean titer; *CI*, confidence interval; *OR*: odds ratio

*Adjusted by age, gender, BMI, marital status, education level, influenza vaccination history, smoking, drinking, and chronic diseases

### Logistic regression analysis of factors influencing the SARS-CoV-2 neutralizing antibody immunization

Logistic regression analysis results showed that only vaccination regimen was associated with SARS-CoV-2 neutralizing antibody immunization. After adjusting for age, gender, BMI, marital status, education level, influenza vaccination history, smoking, drinking, and chronic diseases, the results showed that the participants in 0–21 and 0–28 groups were 4.1 (95% *CI*: 1.9–8.8) and 3.6 (95% *CI*: 1.7–7.6) times more likely to be positive (GMT ≥ 32) than those in 0–14 groups, respectively; and the participants in 0–21 and 0–28 groups showed higher positive rates than those in 0–14 group (GMT ≥ 64) (*OR*: 3.5, 95% *CI*: 2.3–5.4; *OR*: 2.7, 95% *CI*: 1.8–4.1). The similar results were found at GMT ≥ 128 or 256 (Table 4).

**Table.4:** Logistic regression analysis of factors influencing the SARS-CoV-2 neutralizing antibody immunization

Variables	*OR* (95% *CI*)	*OR* (95% *CI*)*
GMT ≥ 32		
0–14 group	1.0	1.0
0–21 group	4.1(1.9–8.6)	4.1(1.9–8.8)
0–28 group	3.5(1.7–7.3)	3.6(1.7–7.6)
GMT ≥ 64		
0–14 group	1.0	1.0
0–21 group	3.5(2.3–5.4)	3.5(2.3–5.4)
0–28 group	2.7(1.8–4.1)	2.7(1.8–4.1)
GMT ≥ 128		
0–14 group	1.0	1.0
0–21 group	2.7(1.9–3.9)	2.7(1.9–3.9)
0–28 group	2.3(1.6–3.3)	2.3(1.6–3.4)
GMT ≥ 256		
0–14 group	1.0	1.0
0–21 group	1.8(1.0–3.1)	1.8 (1.0–3.2)
0–28 group	2.1(1.2–3.7)	2.3(1.3–4.0)

*GMT* geometric mean titer; *CI* confidence interval; *OR* odds ratio

*Adjusted by age, gender, BMI, marital status, education level, influenza vaccination history, smoking, drinking, and chronic diseases

### Safety outcomes

The overall incidence of adverse reactions was 4.1% (11/270), 4.8% (13/270), and 3.7% (10/269) in the 0–14, 0–21 and 0–28 vaccination cohort group.

Solicited adverse reactions were reported by 8 (3.0%) in the 0–14 vaccination cohort group, 11 (4.1%) in the 0–21 vaccination cohort group, and 7 (2.6%) in the 0–28 vaccination cohort group within 7 days after injection. No significant differences were found in the occurrence of solicited and unsolicited adverse reactions among the three groups. Pain, swelling, pruritus, diarrhea and fatigue within 7 days after vaccination were reported by 4 (1.5%), 2 (0.7%), 1 (0.4%), 0 (0.0%), and 1 (0.4%) subject in the 0–14 group, 7 (2.6%), 0 (0.0%), 2 (0.7%), 1 (0.4%), and 1 (0.4%) subject in the 0–21 group, and 2 (0.7%), 2 (0.7%), 0 (0.0%), 1 (0.4%), and 2 (0.7%) subject in the 0–28 vaccination group, respectively. Rash, cough and headache within 28 days after vaccination were reported by 1 (0.4%), 1 (0.4%), and 1 (0.4%) subject in the 0–14 group, 1 (0.4%), 1 (0.4%), and 0 (0.0%) subject in the 0–21 group, and 1 (0.4%), 1 (0.4%), and 1 (0.4%) subject in the 0–28 group, respectively. The reported adverse reactions did not differ significantly among the three study groups (*P* > 0.05). None of the subjects reported serious adverse reactions or became SARS-CoV-2 infected during the follow-up period (Table 5).

**Table.5:** Summary of solicited and unsolicited adverse reactions occurred within 28 days during the study period

Adverse reaction^*^	0–14 group (*n* = 270)	0–21 group (*n* = 270)	0–28 group (*n* = 269)	*P*
Solicited adverse reactions within 0–7 days				
Local reactions	7 (2.6)	9 (3.3)	4 (1.5)	0.418
Pain	4 (1.5)	7 (2.6)	2 (0.7)	0.263
Swelling	2 (0.7)	0 (0.0)	2 (0.7)	0.479
Pruritus	1 (0.4)	2 (0.7)	0 (0.0)	0.777
Systemic reactions	1 (0.4)	2 (0.7)	3 (1.1)	0.545
Diarrhea	0 (0.0)	1 (0.4)	1 (0.4)	0.777
Fatigue	1 (0.4)	1 (0.4)	2 (0.7)	0.702
Unsolicited adverse reactions within 8–28 days				
Local reactions	1 (0.4)	1 (0.4)	1(0.4)	1.000
Rash	1 (0.4)	1 (0.4)	1(0.4)	1.000
Systemic reactions	2 (0.7)	1 (0.4)	2(0.7)	0.876
Cough	1 (0.4)	1 (0.4)	1(0.4)	1.000
Headache	1 (0.4)	0 (0.0)	1(0.4)	0.777

Results expressed as *n* (%)

*Adverse reaction data only list the occurrence of this symptom

## Discussion

Given the COVID-19 pandemic continuing to unfold, a safe and effective vaccine is necessary to contain the global COVID-19 pandemic and prevent further illness and fatalities. Inactivated vaccines are generally safe and widely used for prevention of infectious diseases. The airport ground staff and public security officers are in close contact with other personnel and face greater occupational risk exposure to SARS-CoV-2 infection. Although SARS-CoV-2 vaccines are widely administered in China or other countries, the optimal interval of injections remains unclear and there is lack of randomized controlled trials of inactivated SARS-CoV-2 vaccine in high-risk occupational population. This is the first randomized controlled trial for assessment of the immunogenicity and safety of an inactivated SARS-CoV-2 vaccine in high-risk occupational population.

Here, we explored the immunogenicity and safety of the three different SARS-CoV-2 inactivated vaccination schemes, and found that the GMT of neutralizing antibody were between 98.4 and 145.5, with the seroconversion rates (GMT ≥ 16) being 100% in the three groups. The current clinical trials have also assessed the immunogenicity of SARS-CoV-2 vaccines, and have seen comparable results. The existing inactivated virus vaccines have shown significant immune responses (79–100%) and neutralizing antibody titers (18.9–282.7) [12, 13, 16, 18–20]. In addition, the efficacy of other kinds of SARS-CoV-2 vaccines in phase 3 clinical trials is approximately 70.4–95.0% [22–25]. These studies indicated that the current SARS-CoV-2 vaccines have relatively good immunogenicity.

In our research, the SARS-CoV-2 neutralizing antibody titers of the 0–28, 0–21 groups were significantly greater than that of 0–14 group. Xia et al. [12, 13] in both phase 1 and 2 found that a longer interval (21 days and 28 days) produced higher antibody responses compared with a shorter interval schedule (14 days) [GMT: 282.7 (221.2–361.4); 218.0 (181.8–261.3) vs 169.5 (132.2–217.1)]. Similarly, Zhang et al. [16] and Pan et al. [18] found that the 0–28 regimen of inactivated SARS-CoV-2 vaccine induced higher SARS-CoV-2 neutralizing antibody titers and seroconversion rates compared with the 0–14 regimen. All of above studies indicated that longer interval schedule (0–21 regimen or 0–28 regimen) of the inactivated SARS-CoV-2 vaccines may induce a better immunogenicity.

The incidence of adverse reactions in the 0–14, 0–21 and 0–28 groups were similarly low. Moreover, we did not find severe adverse reaction, with the most common symptom being injection-site pain, indicating no safety concerns. The overall incidence of adverse events after vaccination was 3.7–4.8% in our vaccine-treated groups, which is noticeably lower than that of other SARS-CoV-2 vaccine platform candidates such as viral-vectored vaccines, DNA or RNA vaccines [25–30]. The safety profile of this vaccine in our study is also lower than that of other inactivated SARS-CoV-2 vaccines [12, 16], which may be related to different population characteristics, and minor adverse reactions that are not reported.

As the COVID-19 pandemic continues to spread, multiple SARS-COV-2 variants have emerged. Of these, the Delta variant has currently become the dominant strain of SARS-CoV-2, causing public concern around the world [31]. One study of 366 participants aged 18–59 years in Guangzhou found that the two-dose scheme of the inactivated vaccines yielded an overall vaccine effectiveness of 59.0% against the Delta variant infection in real-world settings [32]. A real-world study [33] by Hu et al. found that Delta variant-infected patients in Jiangsu who received two doses of inactivated vaccine had an 88% reduced risk in progressing to the severe stage. Another real-world study [34] in Guangdong also showed that the effectiveness of full inactivated COVID-19 vaccination against COVID-19 pneumonia and severe illness caused by the B.1.617.2 (Delta) variant was 69.5% and 100.0% respectively. These studies indicated that the inactivated vaccines were effective against the Delta variant. Our study only tested the traditional SARS-CoV-2 strain which is one of our limitations.

In addition, our study had several limitations. First, since the majority of public security officers are male, there may be insufficient representation of the population. And we only reported immune response data for the high-risk occupational population aged 18 to 59 years. Further studies are required to assess the immunogenicity of inactivated SARS-CoV-2 vaccine in various populations, including general population, older people, children and adolescents. Second, data on long-term immunogenicity is not yet available, and the ongoing trial will provide more information. Third, cellular immunity and immune memory were not measured in the current study which need to be further studied.

## Conclusions

In summary, a two-dose of inactivated SARS-CoV-2 vaccine at 0–21 days and 0–28 days regimens significantly improved SARS-CoV-2 neutralizing antibody level compared to the 0–14 days regimen in high-risk occupational population, with seroconversion rates of 100.0%, which to some extent provided a basis for optimizing the immunization strategy of inactivated vaccine against COVID-19 among high-risk occupational population. The results were interim and the long-term immunogenicity and actual protection needs further study.

## Supplementary Information

**Additional file 1: Supplement 1.** Demographic and Behavioral Characteristics of High-risk Occupational Population 28 Days after the Whole Course of Vaccination. **Supplement 2.** SARS-CoV-2 Neutralizing Antibody Immunization 28 Days after the Whole Course of Vaccination Stratified by Age and Gender. b. SARS-CoV-2 neutralizing antibody immunization by gender. **Supplement 3.** GMT of SARS-CoV-2 Neutralizing Antibody 28 Days after the Whole Course of Vaccination Stratified by Age and Gender. **Supplement 4.** Distribution of SARS-CoV-2 Neutralizing Antibody 28 Days after the Whole Course of Vaccination Stratified by Age and Gender

## Data Availability

The full study protocol and the datasets, which includes all data fields reported in this study, are available, following manuscript publication, upon request from the corresponding author (Professor Suping Wang, supingwang@sxmu.edu.cn), following the provision of ethics approval.

## Abbreviations

SARS-CoV-2: Severe acute respiratory syndrome coronavirus 2
COVID-19: Coronavirus disease 2019
GMT: Geometric mean titer
*CI*: Confidence interval
ITT: Intention-to-treat
PP: Per-protocol
ARDS: Acute respiratory distress syndrome
BMI: Body mass index
RT-PCR: Reverse transcriptase-polymerase chain reaction
CDC: Center for Disease Control and Prevention
WHO: World Health Organization
