# Supplementary material for: Safety and immunogenicity of inactivated SARS-CoV-2 vaccine in high-risk occupational population: a randomized, parallel, controlled clinical trial
Source: Infect Dis Poverty. 2021 Dec 22;10:138. doi: 10.1186/s40249-021-00924-2 (PMC8692079; doi:10.1186/s40249-021-00924-2)
Supplement: Supplementary file 1 — Additional file 1: Supplement 1. Demographic and Behavioral Characteristics of High-risk Occupational Population 28 Days after the Whole Course of Vaccination. Supplement 2. SARS-CoV-2 Neutralizing Antibody Immunization 28 Days after the Whole Course of Vaccination Stratified by Age and Gender. b. SARS-CoV-2 neutralizing antibody immunization by gender. Supplement 3. GMT of SARS-CoV-2 Neutralizing Antibody 28 Days after the Whole Course of Vaccination Stratified by Age and Gender. Supplement 4. Distribution of SARS-CoV-2 Neutralizing Antibody 28 Days after the Whole Course of Vaccination Stratified by Age and Gender [file 40249_2021_924_MOESM1_ESM.docx]

**Supplement 1** Demographic and Behavioral Characteristics of High-risk Occupational Population 28 Days after the Whole Course of Vaccination

| Characteristics | Total (*n* = 744) | 0-14 group (*n* = 256) | 0-21 group (*n* = 247) | 0-28 group(*n* = 241) | *P* |
| --- | --- | --- | --- | --- | --- |
| Gender |  |  |  |  | 0.493 |
| Male | 538(72.3) | 179 (69.9) | 179(72.5) | 180(74.7) |  |
| Female | 206(27.7) | 77(30.1) | 68(27.5) | 61(25.3) |  |
| Age(year) |  |  |  |  | 0.409 |
| < 40 | 427(57.4) | 139(54.3) | 143(57.9) | 145(60.2) |  |
| ≥ 40 | 317(42.6) | 117(45.7) | 104(42.1) | 96(39.8) |  |
| Education level |  |  |  |  | 0.222 |
| Junior high school or lower | 70(9.4) | 31(12.1) | 23(9.3) | 16(6.6) |  |
| Senior high school | 33(4.4) | 10(3.9) | 9(3.6) | 14(5.8) |  |
| College or higher | 641(86.2) | 215(84.0) | 215(87.1) | 211(87.6) |  |
| Ethnicity |  |  |  |  | 0.481^a^ |
| Han ethnicity | 733(98.5) | 254 (99.2) | 242(98.0) | 237(98.3) |  |
| other | 11(1.5) | 2(0.8) | 5(2.0) | 4(1.7) |  |
| Marital status |  |  |  |  | 0.161 |
| Married | 573(77.0) | 205(80.1) | 179(72.5) | 189(78.5) |  |
| Unmarried | 150(20.2) | 45(17.6) | 62(25.1) | 43(17.8) |  |
| Divorced or widowed | 21(2.8) | 6(2.3) | 6(2.4) | 9(3.7) |  |
| BMI (kg/m^2^) |  |  |  |  | 0.858 |
| < 18.5 | 19(2.6) | 8(3.1) | 6(2.4) | 5(2.1) |  |
| 18.5 – | 308(41.4) | 107(41.8) | 106(42.9) | 95(39.4) |  |
| ≥ 24 | 417(56.0) | 141(55.1) | 135(54.7) | 141(58.5) |  |
| Influenza vaccination history |  |  |  |  | 0.930 |
| No | 497(66.8) | 169(66.0) | 167(67.6) | 161(66.8) |  |
| Yes | 247(33.2) | 87(34.0) | 80(32.4) | 80(33.2) |  |
| Occupation |  |  |  |  | 0.932 |
| Public security officers | 362(48.7) | 125(48.8) | 122(49.4) | 115(47.7) |  |
| Airport ground staff | 382(51.3) | 131(51.2) | 125(50.6) | 126(52.3) |  |
| Smoking |  |  |  |  | 0.227 |
| No | 501(67.3) | 182(71.1) | 165(66.8) | 154(63.9) |  |
| Yes | 243(32.7) | 74(28.9) | 82(33.2) | 87(36.1) |  |
| Drinking |  |  |  |  | 0.960 |
| No | 568(76.3) | 197(77.0) | 188(76.1) | 183(75.9) |  |
| Yes | 176(23.7) | 59(23.0) | 59(24.0) | 58(24.1) |  |
| Chronic diseases |  |  |  |  | 0.954 |
| No | 689(92.6) | 238(93.0) | 229(92.7) | 222(92.1) |  |
| Yes | 55(7.4) | 18(7.0) | 18(7.3) | 19(7.9) |  |

Results expressed as n (%); ^a^ Fisher’s exact test.

**Supplement 2** SARS-CoV-2 Neutralizing Antibody Immunization 28 Days after the Whole Course of Vaccination Stratified by Age and Gender

a. SARS-CoV-2 neutralizing antibody immunization by age

| SARS-CoV-2 neutralizing antibody | age<40 | | |  | age≥40 | | |
| --- | --- | --- | --- | --- | --- | --- | --- |
|  | 0-14 group  (*n* = 139) | 0-21 group  (*n* = 143) | 0-28 group  (*n* = 145) |  | 0-14 group  (*n* = 117) | 0-21 group  (*n* = 104) | 0-28 group  (*n* = 96) |
| GMT ≥ 32 | |  |  |  |  |  |  |
| No n(%) | 25(18.0) | 5(3.5) | 7(4.8) |  | 9(7.7) | 4(3.8) | 3(3.1) |
| Yes n(%) | 114(82.0) ^a^ | 138(96.5) ^b^ | 138(95.2) |  | 108(92.3) | 100(96.2) | 93(96.9) |
| GMT ≥ 64 | |  |  |  |  |  |  |
| No n(%) | 54(38.8) | 19(13.3) | 35(24.1) |  | 46(39.3) | 19(18.3) | 11(11.5) |
| Yes n(%) | 85(61.2) ^a^ | 124(86.7) ^b^ | 110(75.9) ^a^ |  | 71(60.7) ^a^ | 85(81.7) ^b^ | 85(88.5) ^b^ |
| GMT ≥ 128 | |  |  |  |  |  |  |
| No n(%) | 96(69.1) | 65(45.4) | 74(51.0) |  | 79(67.5) | 44(42.3) | 43(44.8) |
| Yes n(%) | 43(30.9) ^a^ | 78(54.6) ^b^ | 71(49.0) ^b^ |  | 38(32.5) ^a^ | 60(57.7) ^b^ | 53(55.2) ^b^ |
| GMT ≥ 256 | |  |  |  |  |  |  |
| No n(%) | 122(87.8) | 124(86.7) | 121(83.5) |  | 111(94.9) | 86(82.7) | 78(81.2) |
| Yes n(%) | 17(12.2) | 19(13.3) | 24(16.5) |  | 6(5.1) ^a^ | 18(17.3) ^b^ | 18(18.8) ^b^ |

b. SARS-CoV-2 neutralizing antibody immunization by gender

| SARS-CoV-2 neutralizing antibody | male | | |  | female | | |
| --- | --- | --- | --- | --- | --- | --- | --- |
|  | 0-14 group  (*n* = 179) | 0-21 group  (*n* = 179) | 0-28 group  (*n* = 180) |  | 0-14 group  (*n* = 77) | 0-21 group  (*n* = 68) | 0-28 group  (*n* = 61) |
| GMT ≥ 32 | |  |  |  |  |  |  |
| No n(%) | 23(12.8) | 5(2.8) | 6(3.3) |  | 11(14.3) | 4(5.9) | 4(6.6) |
| Yes n(%) | 156(87.2) ^a^ | 174(97.2) ^b^ | 174(96.7) |  | 66(85.7) | 64(94.1) | 57(93.4) |
| GMT ≥ 64 | |  |  |  |  |  |  |
| No n(%) | 70(39.1) | 25(14.0) | 35(19.4) |  | 30(39.0) | 13(19.1) | 11(18.0) |
| Yes n(%) | 109(60.9) ^a^ | 154(86.0) ^b^ | 145(80.6) |  | 47(61.0) ^a^ | 55(80.9) | 50(82.0) ^b^ |
| GMT ≥ 128 | |  |  |  |  |  |  |
| No n(%) | 123(68.7) | 76(42.5) | 88(48.9) |  | 52(67.5) | 33(48.5) | 29(47.5) |
| Yes n(%) | 56(31.3) ^a^ | 103(57.5) ^b^ | 92(51.1) ^b^ |  | 25(32.5) ^a^ | 35(51.5) | 32(52.5) ^b^ |
| GMT ≥ 256 | |  |  |  |  |  |  |
| No n(%) | 163(91.1) | 153(85.5) | 151(83.9) |  | 70(90.9) | 57(83.8) | 48(78.7) |
| Yes n(%) | 16(8.9) | 26(14.5) | 29(16.1) |  | 7(9.1) | 11(16.2) | 13(21.3) |

^a b^ There was significant difference with the different letters.

**Supplement 3** GMT of SARS-CoV-2 Neutralizing Antibody 28 Days after the Whole Course of Vaccination Stratified by Age and Gender

a. GMT of SARS-CoV-2 neutralizing antibody by age b. GMT of SARS-CoV-2 neutralizing antibody by gender

**Supplement 4** Distribution of SARS-CoV-2 Neutralizing Antibody 28 Days after the Whole Course of Vaccination Stratified by Age and Gender

a. Distribution of SARS-CoV-2 neutralizing antibody by age b. Distribution of SARS-CoV-2 neutralizing antibody by gender
